# Pneumonia hospitalisation and case-fatality rates in older Australians with and without risk factors for pneumococcal disease: implications for vaccine policy

**DOI:** 10.1017/S0950268818003473

**Published:** 2019-03-01

**Authors:** S. Dirmesropian, B. Liu, J. G. Wood, C. R. MacIntyre, P. McIntyre, S. Karki, S. Jayasinghe, A. T. Newall

**Affiliations:** 1 School of Public Health and Community Medicine, University of New South Wales, Sydney, NSW, Australia; 2 National Centre for Immunisation Research and Surveillance of Vaccine Preventable Diseases (NCIRS), Kids Research Institute, Children's Hospital at Westmead, NSW, Australia; 3 Discipline of Child and Adolescent Health, Sydney Medical School, Sydney, Australia

**Keywords:** Australia, CAP, elderly, high risk, pneumococcal, pneumonia

## Abstract

Community-acquired pneumonia (CAP) results in substantial numbers of hospitalisations and deaths in older adults. There are known lifestyle and medical risk factors for pneumococcal disease but the magnitude of the additional risk is not well quantified in Australia. We used a large population-based prospective cohort study of older adults in the state of New South Wales (45 and Up Study) linked to cause-specific hospitalisations, disease notifications and death registrations from 2006 to 2015. We estimated the age-specific incidence of CAP hospitalisation (ICD-10 J12-18), invasive pneumococcal disease (IPD) notification and presumptive non-invasive pneumococcal CAP hospitalisation (J13 + J18.1, excluding IPD), comparing those with at least one risk factor to those with no risk factors. The hospitalised case-fatality rate (CFR) included deaths in a 30-day window after hospitalisation. Among 266 951 participants followed for 1 850 000 person-years there were 8747 first hospitalisations for CAP, 157 IPD notifications and 305 non-invasive pneumococcal CAP hospitalisations. In persons 65–84 years, 54.7% had at least one identified risk factor, increasing to 57.0% in those ⩾85 years. The incidence of CAP hospitalisation in those ⩾65 years with at least one risk factor was twofold higher than in those without risk factors, 1091/100 000 (95% confidence interval (CI) 1060–1122) compared with 522/100 000 (95% CI 501–545) and IPD in equivalent groups was almost threefold higher (18.40/100 000 (95% CI 14.61–22.87) *vs.* 6.82/100 000 (95% CI 4.56–9.79)). The CFR increased with age but there were limited difference by risk status, except in those aged 45 to 64 years. Adults ⩾65 years with at least one risk factor have much higher rates of CAP and IPD suggesting that additional risk factor-based vaccination strategies may be cost-effective.

## Introduction

Community-acquired pneumonia (CAP) is a frequent cause of hospitalisation in older adults with a substantial proportion of the burden caused by *Streptococcus pneumoniae* [1–4]. In the USA, over 1 million individuals are hospitalised with CAP annually (the majority of whom are aged ⩾65 years) [5] and similarly large numbers are estimated in older Australians on a per-population basis [6], although the proportion attributable to *S. pneumoniae* has been declining [7]. The 30-day mortality rate among ⩾65 years patients hospitalised with CAP has been estimated as 12.5% in the USA [8] and 11.1% in Australia [6].

It is known that both CAP hospitalisation and mortality increase with age and with the presence of various comorbidities and risk behaviours [9, 10] including: heart disease, immunosuppressive treatment, asthma and diabetes, lung disease, alcoholism, cigarette smoking and excessive weight gain [8, 11, 12]. Although individuals with these factors are classified as being at increased risk of pneumococcal disease in Australia and other countries [13, 14], the limited age-specific data on the relative incidence of CAP hospitalisation and mortality risk among such individuals makes it difficult to determine the merits of additional prevention targeted at high-risk groups.

In Australia, the Pharmaceutical Benefits Advisory Committee recently recommended 13-valent pneumococcal conjugate vaccine (PCV13) as a replacement for the existing 23-valent pneumococcal polysaccharide vaccine (PPV23) for all adults aged 65 years and over [15]. In addition to protecting against serotype specific invasive pneumococcal disease (IPD), PCV13 has been found to be effective against vaccine type pneumococcal CAP in older adults [16]. There is a need to quantify the risk of pneumococcal CAP and IPD in high risk groups to help evaluate targeted vaccination strategies (e.g. potential use of PPV23 following PCV13 vaccination [16] in high-risk subgroups).

In order to assess the impact of risk factors on pneumococcal-related diseases, we used a large population-based cohort study of older Australian adults (The Sax Institute's 45 and Up Study [17]) with detailed information on comorbidities linked to hospitalisation, disease notifications and death data. The primary aim was to estimate the incidence of CAP hospitalisations and associated deaths (case-fatality rate, CFR) in relation to known risk factors for pneumococcal-related disease. We also sought to calculate relative risks (RR) for these events in individuals with at least one identified risk factor compared to those without.

## Methods

The Sax Institute's 45 and Up Study is a population based prospective cohort study conducted in New South Wales (NSW) Australia. It consists of approximately 267 000 participants aged ⩾45 years at enrolment. Participants were randomly selected from the Department of Human Services (formerly Medicare Australia) enrolment database between 1 January 2006 and 31 December 2008. A baseline questionnaire was used to collect data on demographics, social characteristics, personal health behaviours and general health-related data, with written consent obtained for data linkage of participants questionnaire information to administrative health databases. Further details of the 45 and Up Study are published [17] and available on the study website (https://www.saxinstitute.org.au/our-work/45-up-study/).

For each individual enrolled, the baseline data were linked to various datasets by the Centre for Health Record Linkage (CHeReL) using probabilistic matching [18]. Datasets relevant to this study included hospitalisation data (both private and public), from the NSW Admitted Patient Data Collection (APDC), disease notifications, from the Notifiable Conditions Information Management System (NCIMS) dataset and cancer and death registrations from the NSW Cancer Registry and Registry of Births, Deaths and Marriages respectively.

The APDC dataset contains records of all hospitalisation events in NSW with the principal and up to 49 secondary diagnoses coded using the International Classification of Diseases 10th Revision, Australian Modification (ICD-10-AM). It also contains data on admission and discharge dates. Data were available from 1 July 2001 until 30 June 2015. The NCIMS dataset contains all notifications of certain infectious diseases (including IPD onset dates) and adverse events following immunisation required under the Public Health Act 2010 [19]. The NSW Cancer Registry contains details for people diagnosed or treated with cancer in NSW and the date of diagnosis since 1972. The Registry of Births, Deaths and Marriages contains records of all deaths occurring in NSW and includes date of death.

### Outcome ascertainment

The main outcomes investigated were incidence rates of first CAP hospitalisation following study enrolment and CFR following hospitalisation for CAP. We also examined various groupings deemed likely to represent non-invasive pneumococcal CAP and IPD notifications. Episodes of CAP were identified if there was a linked hospital record where the principal diagnosis field had ICD-10-AM codes J12–J18, whereas for identification of presumptive pneumococcal CAP, only codes J13 (pneumococcal pneumonia) and J18.1 (lobar pneumonia) were used [20]. Presumptive non-invasive pneumococcal CAP hospitalisations were calculated by removing any linked IPD cases derived from NCIMS data from all cases identified as presumptive pneumococcal CAP using ICD coded hospitalisation data (J13 + J18.1). The NCIMS data capture IPD cases through laboratory notification of *S. pneumoniae* identified at a sterile site. Only hospital admissions and notifications that occurred after enrolment in the 45 and Up Study were included. We also excluded those with a CAP hospitalisation 30 days prior to recruitment to ensure events post-recruitment (up to 30 days) were not re-hospitalisations. Deaths from CAP were identified if a participant had a death registration within 30 days following a CAP hospital separation.

### Identification of risk groups

We defined risk groups for pneumococcal-related disease a priori according to the Australian Immunisation Handbook [13] as these groups may be considered eligible for alternative vaccination approaches (e.g. additional dose/s). In our primary analysis we compared those where at least one risk category was identified (‘any risk’) with those where no risk factor was identified (‘no-risk’). Risk categories included: alcoholism, smoking, chronic heart disease, diabetes, asthma, chronic lung disease, chronic liver disease, chronic renal failure, asplenia, immuno-suppressive conditions (including haematologic cancer) and other malignancies (excluding non-melanoma skin cancer) [13].

Alcoholism, smoking, heart disease, diabetes and asthma were identified based on self-report in the 45 and Up Study recruitment questionnaire. Risky alcohol consumption (a proxy for alcoholism) was defined as drinking >28 drinks per week for men and >14 drinks per week for women based on Australian guidelines [21]. Current smoking was classified as those who answered yes to ever smoking and indicated they were current smokers. The presence of heart disease, diabetes and asthma was based on self-report of being diagnosed with the relevant condition by a doctor.

Non-cancer immunosuppressive conditions, renal disease, chronic respiratory disease (requiring hospitalisation, including asthma) and liver disease were identified based on having a linked APDC hospital admission record with a relevant ICD-10-AM diagnosis code prior to recruitment as first (principal) or second (diagnosis_code1) diagnosis field (see supplementary Appendix 1 Table S1). The haematological disorders included non-cancer haematological conditions and haematological cancers. The haematological cancer and other cancers group were defined as having a linked cancer registry record with a relevant ICD-10 diagnosis code and a diagnosis date before recruitment (see supplementary Appendix 1 Table S1).

### Statistical analysis

For demographic analyses, we stratified participants into the age groups of 45–64, 65–74, 75–84 and ⩾85 years based on their age at recruitment. However, for incidence and fatality rates, the attained age at event (hospitalisation or death) was used. For each age strata we further disaggregated participants by risk group before calculating CAP hospitalisation incidence and CFRs. Where data were missing for one risk factor and no other risk factors were present, the subjects were categorised into a ‘missing data’ group and included in analyses. Due to the small numbers of IPD cases and hospitalisations specifically coded as pneumococcal CAP, for reporting IPD and presumptive pneumococcal CAP hospitalisation rates for each age strata we combined all risk factors into one single ‘any risk’ group (those with at least one of the pre-defined risk factors).

Since most risk factor information was only available at baseline, the hospitalisation incidence was calculated by dividing the number of first episodes since recruitment by the person-years at risk [22]. Using first events avoids repeat hospitalisation for a single event and the potential for a first hospitalisation to influence the future risks for the same event. Person-years at risk was defined as the period from enrolment until 30 June 2015 (the latest date for which complete hospital data were available), or the date of the first hospitalisation record for CAP, or the date of death, whichever occurred first. Similarly, to calculate IPD rates the period until the first notification record since recruitment was divided by the person-years at risk.

For calculation of the CFR of hospitalised CAP, the number of deaths in the 30-day time window after separation was divided by the total number of CAP hospitalisations for that group. It is important to note that for incidence of CAP hospitalisation, only the first episode of CAP hospitalisation for each subject was used to estimate incidence, whereas for the CFR calculation, deaths after any CAP hospitalisation episode (first or subsequent) were included.

All data preparation including identification and extraction of risk groups, calculation of incidence and CFR were completed in the R programming language. The R packages *Epi* and *Epitools* were used to calculate person years at risk by age group and confidence intervals around estimates outcomes respectively.

## Results

Our total study population included 266 951 participants of which 53.6% were female. At recruitment, 61.3% were aged 45–64 years, 21.7% were 65–74 years, 13.8% were 75–84 years and 3.1% were aged 85 and over. Among participants, 44.2% had at least one designated risk factor identified, 52.9% had no risk factors identified and 2.9% had missing data for at least one risk factor. The largest risk group was heart disease (11.9% of participants) followed by non-haematological cancers (10.2%), asthma (10.2%) and diabetes (9%). Further details are provided in Table 1.

**Table 1.** Distribution of risk factors for pneumococcal disease based on age group and sex at baseline in the 45 and Up Study

	Age groups at baseline (in years)											
	45–64			65–74			75–84			⩾85		
	*N*	Prop.	Female (%)	*N*	Prop.	Female (%)	*N*	Prop.	Female (%)	*N*	Prop.	Female (%)
Alcohol	12 786	7.9	51.4	3505	6.2	39.7	1259	3.5	38.4	157	2.0	58.6
Smoking	15 429	9.5	53.4	2828	4.9	42.0	928	2.5	40.6	136	1.7	53.7
Heart disease	9966	6.1	38.0	9897	17.0	34.7	9635	26.1	34.6	2255	27.3	49.8
Diabetes	10 564	6.4	46.6	7309	12.6	40.2	5122	13.8	39.6	936	11.3	52.0
Asthma	17 300	10.8	65.1	5740	10.1	59.0	3479	9.6	53.8	638	7.7	63.3
Haematological disorders including cancers	916	0.6	63.0	450	0.8	50.7	406	1.0	41.0	78	0.9	55.1
Non-haematological cancer	10 103	6.2	59.8	8512	14.7	39.6	7139	19.3	31.9	1511	18.3	41.2
Renal disease	1204	0.7	46.0	762	1.3	34.9	796	2.2	32.8	195	2.4	36.9
Chronic respiratory diseases	1704	1.0	64.9	1418	2.4	48.5	1591	4.3	43.0	469	5.7	53.0
Chronic liver diseases	607	0.4	44.1	194	0.3	38.7	104	0.3	38.5	16	0.2	75.0
Any risk factor	62 916	39.6	57.0	29 241	51.7	43.1	21 226	59.3	38..9	4525	57.0	50.8
No risk factors	95 982	60.4	58.1	27 294	48.3	54.6	14 541	40.7	53.5	3409	43.0	61.2
All participants	158 898	100	56.9	56 538	100	49.1	35 767	100	45.4	7934	100	55.8

*N*, total number of that age group; Prop, proportion of total number of that age group.

**Fig. 1.**
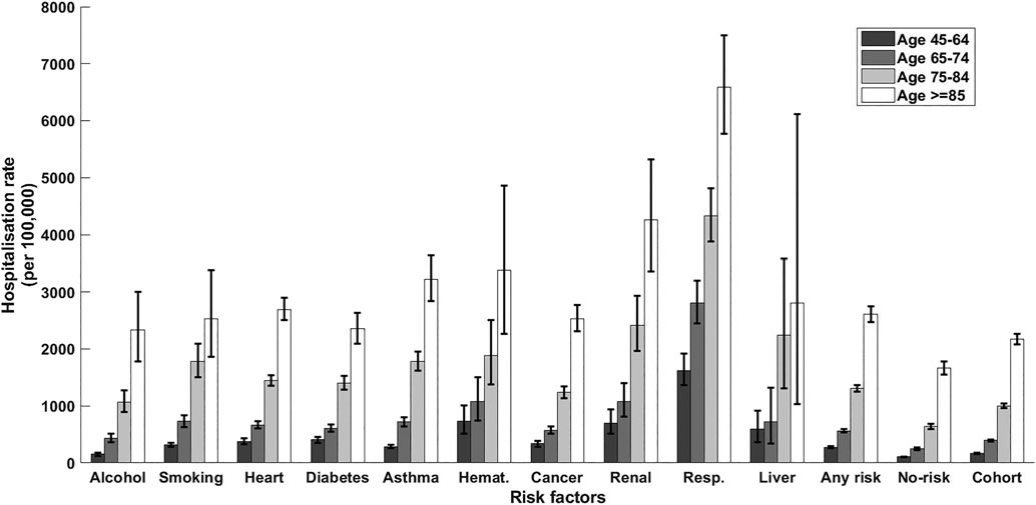
CAP hospitalisation rate stratified by risk factor and age group. Error bars = 95% CIs; haemat., immunosuppressive conditions due to haematologic problems or haematologic cancer; cancer, all other non-haematologic cancers; resp., chronic respiratory diseases; no-risk, none of risk factors identified; any risk, at least one risk factor identified; cohort, all participants.

**Fig. 2.**
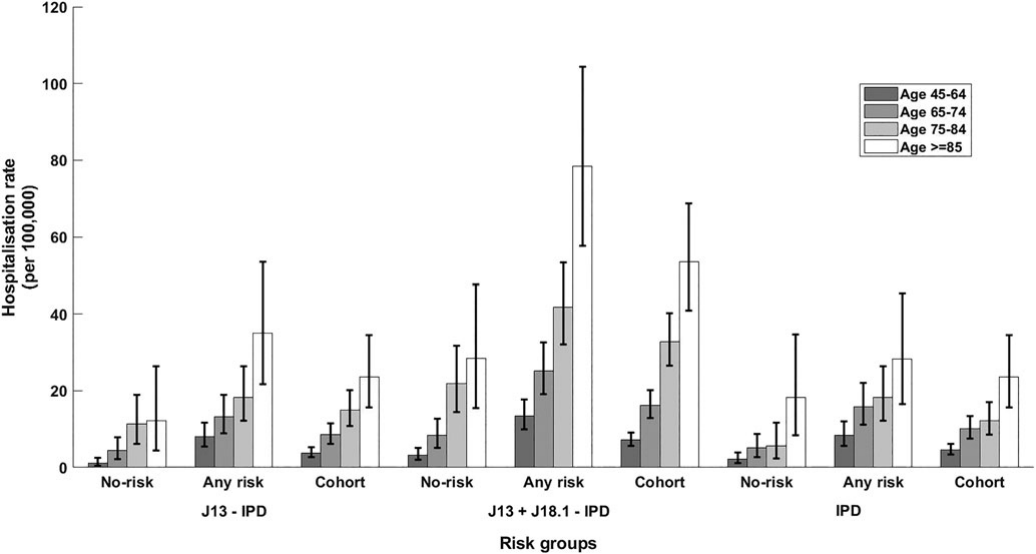
Rate of hospitalisation for different definitions of presumptive non-invasive pneumococcal CAP and rate of IPD notification stratified by presence of risk factors and age group. Error bars = 95% CIs; no-risk, none of risk factors identified; any risk, at least one risk factor identified; cohort, all participants. J13-IPD, ICD coded J13 hospitalisations after removing any with linked IPD notification; J13 + J18.1-IPD, ICD coded J13 or J18.1 hospitalisations after removing any with linked IPD notification; IPD, invasive pneumococcal disease notifications.

Within the cohort, over 1 850 000 person-years, 8747 participants had a CAP hospitalisation, 157 an IPD notification and 305 a presumptive non-invasive pneumococcal CAP hospitalisation. The distribution of first episodes of CAP hospitalisation among risk groups, further stratified by age, is summarised in Table 2. Relevant incidence rates are compared in Figure 1. The incidence of both CAP hospitalisation and IPD notification (Fig. 2) increased with age and, among each age group, was consistently higher in the ‘any risk’ group than the ‘no-risk’ group. For each age group, the CAP incidence rate was highest in those with chronic respiratory disease and lowest in the group with no risk factors (‘no-risk’). In the 65–74 year age group, the incidence of CAP hospitalisation was 563/100 000 (95% confidence interval (CI), 532–595) in those with at least one risk factor compared with 247/100 000 (95% CI 228–267) in those with no risk factors, a RR of 2.28 (95% CI 2.07–2.51). The RR for 75–84 years and ⩾85 years for the same comparison were 2.06 (95% CI 1.90–2.24) and 1.56 (95% CI 1.44–1.70) respectively. Age-specific incidence rates were relatively similar in those with missing data on risk status to the entire cohort.

**Table 2.** Number of first hospitalisations for community acquired pneumonia (CAP) following recruitment and person-years of follow-up stratified by risk factor and age group

	Attained age (in years)							
	45–64		65–74		75–84		⩾85	
	Hosp.	Person years	Hosp.	Person years	Hosp.	Person years	Hosp.	Person years
Alcohol	114	76 072	148	33 969	127	11 906	60	2568
Smoking	304	95 598	207	28 375	145	8139	46	1814
Heart disease	183	48 681	431	64 831	905	62 541	735	27 283
Diabetes	222	55 009	318	52 183	521	37 255	299	12 705
Asthma	286	99 744	329	45 759	439	24 650	258	8020
Haematological disorders including cancer	37	5037	33	3078	47	2497	29	858
Non-haematological cancer	168	50 178	321	56 002	579	46 844	462	18 248
Renal disease	44	6298	56	5175	102	4223	77	1808
Chronic respiratory diseases	138	8519	225	8012	338	7808	229	3474
Chronic liver diseases	20	3365	10	1389	17	760.	6	231
Any risk factor	975	355 667	1257	223 327	1947	149 178	1479	56 707
No risk factors	612	582 128	619	250 530	784	122 039	792	47 546
Participants with missing data on any risk factor group	56	33 525	50	15 268	81	9297	95	4630
All participants	1643	971 319	1926	489 125	2812	280 515	2366	108 884

Hosp, number of hospitalisation of first episode of CAP.

The incidence of IPD notification for the 65–74 years age group was threefold higher (15.88/100 000 (95% CI 11.12–21.99)) in those with at least one risk factor compared with those without (5.15/100 000 (95% CI 2.74–8.81)), resulting in a RR of 3.08 (95% CI 1.63–5.81). The RR for the same comparison within those aged 75–84 years was similar 3.22 (95% CI 1.41–7.38) with incidence rates of 18.24/100 000 (95% CI 12.12–26.36) and 5.65/100 000 (95% CI 2.27–11.65) but the RR was substantially lower in those aged 85+, 1.55 (95% CI 0.69–3.39), with rates of 28.50/100 000 (95% CI 16.52–45.39) and 18.23/100 000 (95% CI 8.33–34.60). The overall IPD incidence rates per 100 000 were 10.11, 12.20 and 23.65 for those aged 65–74, 75–84 and 85+ respectively.

For non-invasive pneumococcal CAP (J13 + J18.1-IPD), we summarise hospitalisation data in Table 3. As shown in Figure 2, the relative incidence of non-invasive pneumococcal CAP also increased with age and by risk status. In the 65–74 years age group, incidence of non-invasive pneumococcal CAP (J13+J18.1-IPD) hospitalisation in the ‘any risk’ group was 25/100 000 (95% CI 19.06–32.60) compared with 8/100 000 (95% CI 5–13) in the ‘no-risk’ group, RR 3.02 (95% CI 1.83–4.98). The RR for ages 75–84 years and ⩾85 years for the same comparison was 1.91 (95% CI 1.22–3.00) and 2.76 (95% CI 1.52–5.02) respectively (Fig. 2).

**Table 3.** Number of hospitalisations based on different definitions of presumptive non-invasive pneumococcal pneumonia and number of IPD notifications stratified by the presence of risk factors and age group

	Age groups											
	45–64			65–74			75–84			⩾85		
	(J13-IPD)^a^	(J13 + J18.1-IPD)^b^	IPD^c^	(J13-IPD)	(J13 + J18.1-IPD)	IPD	(J13-IPD)	(J13 + J18.1-IPD)	IPD	(J13-IPD)	(J13 + J18.1-IPD)	IPD
Any risk factor	29	48	30	30	57	36	28	64	28	21	47	17
No risk factors	7	19	13	11	21	13	14	27	7	6	14	9
All participants	37	70	45	42	80	50	43	94	35	27	61	27

a ICD coded J13 hospitalisations after removing any with linked IPD notification.

b ICD coded J13 or J18.1 hospitalisations after removing any with linked IPD notification.

c Invasive pneumococcal disease notifications.

In the 65–74 age group, the CFR following CAP hospitalisation for those in ‘any risk’ group was 6.6% (95% CI 5.5–8.0) compared with 5.4% (95% CI 3.8–7.3) in the ‘no-risk’ group, a combined CFR of 6.2% (95% CI 5.3–7.3) (Table 4). The CFR within each risk group progressively increased with age but there were limited difference by risk status, except in those aged 45 to 64 years. For instance, in the 85+ years group the CFR was 16.7% (95% CI 15–18.6) in ‘any risk’ group *vs.* 15.1% (95% CI 12.8–17.7) in the ‘no-risk’ group. Persons with heart disease risk group had the highest number of CAP-associated deaths (Table 4), accounting for 30.7% of all deaths following CAP hospitalisation, followed by those with non-haematological cancer, diabetes and chronic obstructive pulmonary disease (COPD).

**Table 4.** Hospitalisation case fatality rate following CAP hospitalisation (30 days after separation) stratified by risk factor and age group

	Age groups (in years)							
	45–64		65–74		75–84		⩾85	
	CFR^a^ %	95% CI^b^	CFR %	95% CI	CFR %	95% CI	CFR %	95% CI
Alcohol	7.09 (10/141)	3.4–13.04	8.33 (15/180)	4.66–13.74	13.76 (26/189)	8.99–20.16	22.97 (17/74)	13.38–36.78
Smoking	5.14 (20/389)	3.14–7.94	8.63 (24/278)	5.53–12.85	12.17 (23/189)	7.71–18.26	23.44 (15/64)	13.12–38.66
Heart disease	5.11 (12/235)	2.64–8.92	4.70 (27/575)	3.09–6.83	9.01 (112/1243)	7.42–10.84	17.31 (175/1011)	14.48–20.07
Diabetes	4.51 (13/288)	2.4–7.72	7.28 (30/412)	4.91–10.39	9.82 (72/733)	7.69–12.37	15.23 (67/440)	11.80–19.34
Asthma	3.00 (11/367)	1.50–5.36	6.43 (27/420)	4.24–9.35	6.66 (40/601)	4.75–9.06	14.58 (56/384)	11.02–18.94
Haematological disorders including cancer	6.90 (4/58)	1.88–17.66	NA^c^	0.55–16.42	12.31 (8/65)	5.31–24.25	23.68 (9/38)	10.83–44.96
Non-haematological cancer	9.09 (20/220)	5.55–14.04	11.01 (47/427)	8.09–14.64	11.20 (85/759)	8.95–13.85	15.48 (102/659)	12.62–18.79
Renal disease	8.20 (5/61)	2.66–19.13	NA	0.31–9.26	11.80 (19/161)	7.11–18.43	14.17 (17/120)	8.25–22.68
Chronic respiratory diseases	3.68 (7/190)	1.48–7.59	7.21 (23/319)	4.57–10.82	9.55 (51/534)	7.11–12.56	14.32 (54/377)	10.76–18.69
Any risk factor^d^	4.96 (62/1250)	3.80–6.36	6.64 (111/1672)	5.46–7.99	9.53 (254/2664)	8.40–10.78	16.75 (349/2084)	15.03–18.60
No risk factors	1.95 (14/718)	1.07–3.27	5.38 (40/744)	3.84–7.32	8.02 (79/985)	6.35–10.00	15.06 (152/1009)	12.76–17.66
All participants	3.84 (78/2030)	3.04–4.80	6.22 (154/2475)	5.28–7.29	9.09 (341/3751)	8.15–10.11	16.35 (527/3223)	14.98–17.81

a Case fatality rate calculated by dividing number of deaths by the number of hospitalisations.

b Confidence intervals.

c NA, not applicable, due to small number of cases (privacy restriction).

d Chronic liver group was not presented as separate group due to small numbers but included in any risk group.

## Discussion

In this study we quantified the incidence and RR of CAP and pneumococcal-related outcomes among pre-defined risk groups in a large cohort of older Australians. Comparing those with at least one risk factor to those without, we found large differences in the incidence of (pneumococcal) CAP hospitalisation and IPD but limited differences in fatality rates following CAP hospitalisation. For example, in those ⩾65 years the incidence of CAP hospitalisation in the ‘any risk’ group was twice that of the rate in the ‘no-risk’ group. However, while the point estimates for CFR by age were always higher in those with at least one risk factor, the differences based on risk status were relatively small and did not reach statistical significance in any of the age groups ⩾65 years. This suggests the CAP risk factors analysed here substantially increase the probability of severe CAP requiring hospitalisation but that once hospitalised they have limited further impact of subsequent mortality outcomes in those aged over 65 years.

The incidence rate of CAP hospitalisation varied significantly within individual risk groups. For instance, in the 65–74 year age group the incidence varied from 2808/100 000 (95% CI 2453–3200) for those with COPD to 436/100 000 (95% CI 368–512) for those in the risky alcohol consumption group. The relatively low incidence in the alcohol risk group could be related to the definition based on alcohol consumption rather than alcoholism. It is important to note that risk groups with high incidence overall sometimes have low impact on total number of CAP hospitalisations. For instance, in the 65–74 age group, the renal disease risk group RR was 4.38 (95% CI 3.37–5.75) compared with no-risk group but due to small numbers of individuals with this risk factor it resulted in only 56 hospitalisations. We did not focus in more detail on individual risk factors as the relevant policy question is around whether to provide additional vaccines to high-risk groups as a whole.

Only a small number of international studies provide outcomes related to CAP risk factors that are comparable with our main outcomes. A US study by Shea *et al*. reported RR for all-cause CAP in ⩾65 age group to be 3.0 (95% CI 3.0–3.0) when the at-risk group was compared with no-risk counterparts [23]. In our analysis the RR for the same age group was 2.09 (95% CI 1.98–2.20). The studies had quite different designs with Shea *et al*., a retrospective analysis of insurance data that included outpatient claims whilst we only examined hospitalisations. Our findings more closely resemble that of a multi-centre prospective German study (that also included outpatient CAP) for those aged over 60 years that reported a RR for all-cause CAP of 2.5 (95% CI 2.4–2.5) in at-risk groups [24]. Many of the other existing studies focused on identifying risk factors in those already hospitalised for CAP. For example, Koivula *et al*. estimated the proportion of those with risk factors among adults hospitalised with CAP and reported that 23.7% and 13.1% of those hospitalised had heart disease and diabetes respectively [12]. In our study heart disease and diabetes were identified in 29.1% and 16% of those hospitalised due to CAP respectively.

There have been a number of studies that have estimated mortality following CAP. Using data from the CAPiTA study, a randomised placebo-controlled trial of PCV13 in the Netherlands, Vissink *et al*. found 30 day mortality rates following CAP hospitalisation of 4% in low risk, 10.4% in medium risk (presence of other chronic conditions) and 29.1% in high risk (i.e. immune-compromised) patients [25]. In one of the few existing Australian studies on pneumonia CFR, a case cohort study [6] by Skull *et al*., the 30 day mortality rate for CAP hospitalisation was estimated to be 18% in those aged ⩾65 years. In comparison, for the ⩾65 year age group, we estimated a CFR of 10.76% following CAP hospitalisation. One potential reason for the differences between studies could be the differences in the populations between the two studies (healthier cohort compared with Average Australian) and in the inclusion criteria for CAP (J12–J18 in our study *vs.* J10–J18 in the study by Skull *et al*.).

Our analysis using the 45 and Up Study prospective cohort has several advantages over previous published studies. It had a large sample size (~265 000 at recruitment) long duration of follow-up (10 years) and linkage to several health datasets. However, there are also several limitations to our study. First, the cohort population is known to be healthier compared with the general population [17], probably due to continuous monitoring of health status of participants and also enrolment bias. Indeed the age-specific CAP hospitalisation rates for the cohort were lower than comparable rates in the Australian population [26]. However, the RRs calculated here may be more transferable to population estimates of risk-based incidence. It should be noted that as we were not attempting to identify groups at risk but rather focused on establishing the (relative) incidence in those defined as at risk [13], we did not undertake adjusted analyses to deal with confounding or estimate population attributable risks for individual risk factors.

Several other limitations in the study relate to the identification of risk factors. In the case of risk factors which are identified through hospitalisation (APDC) data, we only examined data back to 2001 and only used the principal or second diagnosis field, this may underestimate the prevalence of those risk factors. Also, all risk factors were measured only at baseline and we did not examine the change of hospitalisation codes used for identification of risk factors during 14 years of follow-up. Therefore the influence of risk factors that develop following recruitment may lead to some unknown bias in our results. Finally, the identification of pneumococcal CAP was limited by the ICD coding data available, with it likely that the majority of pneumococcal CAP cases are included in the ‘pneumonia not specified’ (ICD code, J18.9) category. A lesser potential bias in presumptive pneumococcal CAP ascertainment in our study is that while lobar pneumonia (ICD code J18.1) is strongly associated with pneumococcal infection [20, 26, 27], some of these cases may be due to other causes.

Older adults with at least one pneumococcal risk factor had substantially higher rates of CAP and pneumococcal-related outcomes. This may make further vaccination targeted at this group cost-effective. For example, if additional dose/s of pneumococcal vaccine were deemed not to be cost-effective in all adults (above a given age) they may still be cost-effective in those with risk factors. However, the immune response to vaccination, particularly in patients with cancer or other causes of immune-suppression, may be sub-optimal and would need to be considered. The results from this study can be used to help inform pneumococcal vaccination policy in high-risk adults both within the Australian context and in comparable international settings.

## Supplementary material

For supplementary material accompanying this paper visit https://doi.org/10.1017/S0950268818003473. click here to view supplementary material
