# Supplementary material for: Pneumonia hospitalisation and case-fatality rates in older Australians with and without risk factors for pneumococcal disease: implications for vaccine policy
Source: Epidemiol Infect. 2019 Mar 1;147:e118. doi: 10.1017/S0950268818003473 (PMC6518507; doi:10.1017/S0950268818003473)
Supplement: Supplementary file 1 [file S0950268818003473sup001.docx]

**Appendix 1**

Table A1-1 List of ICD-10 codes used to define risk groups including non-cancer immunosuppressive conditions, renal disease, chronic respiratory disease and liver disease, haematological disorders (including haematological cancers) and non-haematological cancer

|  | ICD-10 code |
| --- | --- |
| Non-cancer immunosuppressive disease | D56, D57, D58, D59, D73, K90.0 |
| Haematological disorders including cancer | C81, C82, C83, C84, C85, C86, C87, C88, C90, C91, C92, C93, C94, C95, C96 |
| Non-haematological cancers | All cancer records without a haematological cancer code |
| Renal disease | N00 to N08, N10 to N16, N18, N19, N25 to N29, Q60 to Q63 |
| Chronic respiratory disease | J40 to J47, J81, J84, Q30 to Q36 |
| Chronic liver disease | B18, K70 to K77, Q44 |

Table A1-2 Hospitalisation rate for CAP calculated as incidence per 100,000 person years, stratified by risk factor and age group.

|  |  |  |  | Age groups (in years) |  |  |  |  |
| --- | --- | --- | --- | --- | --- | --- | --- | --- |
|  | 45-64 | | 65-74 | | 75-84 | | ≥ 85 | |
|  | Hosp. rate^1^ | CI^2^ 95% | Hosp. rate | CI 95% | Hosp. rate | CI 95% | Hosp. rate | CI 95% |
| Alcohol | 150 | 124-180 | 436 | 368-512 | 1067 | 889-1269 | 2336 | 1783-3007 |
| Smoking | 318 | 283-356 | 730 | 633-836 | 1782 | 1503-2096 | 2536 | 1856-3382 |
| Heart disease | 376 | 323-434 | 665 | 603-731 | 1447 | 1354-1544 | 2694 | 2503-2896 |
| Diabetes | 404 | 352-460 | 609 | 544-680 | 1398 | 1618-1955 | 2353 | 2094-2636 |
| Asthma | 287 | 254-322 | 719 | 643-801 | 1781 | 1155-1356 | 3217 | 2836-3634 |
|  |  |  |  |  |  |  |  |  |
| Haematological disorders including cancer | 735 | 517-1012 | 1072 | 738-1505 | 1882 | 1383-2503 | 3380 | 2263-4854 |
| Non-haematological cancer | 335 | 286-389 | 573 | 512-639 | 1236 | 1137-1341 | 2532 | 2306-2773 |
| Renal disease | 699 | 508-938 | 1082 | 817-1405 | 2415 | 1969-2932 | 4260 | 3362-5324 |
| Chronic respiratory diseases | 1620 | 1361-1914 | 2808 | 2453-3200 | 4329 | 3880-4816 | 6592 | 5765-7503 |
| Chronic liver diseases | 594 | 363-918 | 720 | 345-1323 | 2236 | 1303-3580 | 2812 | 1032-6121 |
| Any risk factor | 274 | 257-292 | 563 | 532-595 | 1305 | 1248-1364 | 2608 | 2477-2744 |
| No risk factors | 105 | 97-114 | 247 | 228-267 | 642 | 598-689 | 1666 | 1552-1786 |
| Participants with missing data on any risk factor group | 167 | 126-217 | 327 | 243-432 | 871 | 692-1083 | 2052 | 1660-2508 |
| All participants | 169 | 161-177 | 394 | 376-411 | 1002 | 966-1040 | 2173 | 2086-2262 |

1 Hospitalisation rate per 100,000 person years

2 Confidence interval
